# Dr. Mossman, on Digitalis, in Reply to Harlestonensis

**Published:** 1808-10

**Authors:** George Mossman

**Affiliations:** Bradford, Yorkshire


### *To the Editors of the Medical and Physical Journal.*

Gentlemen,

In the Fourth Number of your Nineteenth Volume, there is a paper of a very singular complexion, signed *Harlestonensis,*

*tonensis.* The paragraphs which I select for animadversion, are the following:

"The rage for novelty pervades medical society, in common with others; there is a fashion even in medicine; and it would seem, that it matters not, however absurd and preposterous the opinions of some men are, so that they are but new and singular. I am led to this observation from hearing almost daily, different medicines announced, and highly extolled, in the cure of particular complaints, by men high in the medical world, eagerly taken up by others, and, after a trial, as readily abandoned; thus we have seen Digitalis, Uva Ursi, Lichen Islandicus, Oxydum Bismuthi, Dr. Lambe's Distilled Water, and a long catalogue of et ceteras, fall into disrepute."

The present period may justly be denominated the period of enquiry, and I am persuaded, that that spirit claims a more noble origin, than a "rage for novelty;" it springs from an anxious and laudable solicitude to discover the means of alleviating human evil. The medical men, and the medical publications of the present day, are highly respectable; and in the present state of medical literature, a patient investigation of the truth is a feature very justly conspicuous. This is as it should be; for surely an affectation of singularity should never seize upon a mind contemplating subjects inseparably connected with the happiness and the lives of our species.

If medical opinions be the result of clinical observation, and formed on an extended experience, it is insufferable arrogance to say, that they are "absurd and preposterous." I have no knowledge of your anonymous Correspondent, but from his unqualified censure of his medical brethren, and the instruments they employ in the cure of disease, I am persuaded he is a very young man, and very inexperienced. The period of youth is frequently unreasonably opiniative, or unreasonably sceptical. From the walls of a university men frequently come unbelievers in the art of healing, or they possess the vanity of being able to cure every disease. Time and experience will generally correct both those extremes.

The practice of twenty years will leave no doubt on the mind of a physician, that he can alleviate much distress, and it will also teach him that there are many ills which he cannot cure. The pursuit of curative agents should certainly never be abandoned, so long as there remains a disease to be cured. If this observation be correct, it ought to justify the introduction, and the employment of those powers, which *Harlestonensis* has attempted to sweep away. To overawe speculation in any art, or in any science, is to doom into a state, stationary as to improvement, and in the department of medicine, we can never be competent to appreciate the value of *Digatalis*, or any other agent, till we have patiently tried their effects on the system.

*Harlestonensis* may have employed, and he may have abandoned the articles which he has mentioned; but we ought to have his practice more in detail; we ought to be informed how long; in what doses; and in what diseases he employed those agents. Till he put us in possession of additional information, we cannot admit of consigning to oblivion, by one "fell swoop," powers so generally and so beneficially employed. The first article of his reprobation is *Digitalis*. What preparations of the plant? in what doses? and in what diseases has he administered it? Will he tell us that his experience has settled his faith; that the *Digitalis* exerts no influence over the action of the heart? Or will he tell us, that an unguarded employment of it has, in his hands, produced effects highly deleterious? A cure of disease may be sometimes gradually effected by the instruments of our art; and their operation may never become an immediate object of the senses. The operation of *Digitalis* is speedily perceptible; in a few hours sometimes, but very certainly in a few days, its effects on arterial action are clearly demonstrated.

*Harlestonensis* asserts generally, that the *Digitalis* has been tried and abandoned; with the exception of himself, I would ask him, by whom has it been abandoned? Has it fallen into discredit with those men who know in what preparation, in what doses, at what times, and in what diseases, so administer it? The apothecaries of this place, are men highly respectable; for many years I have witnessed their employment of *Digitalis;* and I have often heard them declare, that were they deprived of its aid, they could not, with satisfaction to themselves, pursue their profession. An ample experience of the power of this plant has made it to them inestimable; and they have long been accustomed to rank it with *opium*, the *bark of Cinchona*, and other important essentials of the *Materia Medica*. The periodical publications too, afford us indisputable evidence of its beneficial employment in every quarter of the British empire, and on the continents of Europe and America. The writers too, who have given it celebrity, are men of the first character. The names of

*Withering*,

*Withering*, *Ferriar*, *Beddoes*, *Drake*, *M'Lean*, *Thomas*, and *Hamilton*, are familiar to every man of medical literature. The late venerable *Heberden*, who took lessons at the bed-side for half a century, speaks of *Digitalis* in language singularly expressive. If this mass of evidence be insufficient to convince Harlestonensis that *Digitalis* continues to be extensively employed, he may have additional testimony, by an application to the venders of the plant. I am not wedded to any opinion; nor am I ambitious to support any particular theory. I profess to have nothing in view but to arrive at a knowledge of the truth; and in obedience to this object, I formerly did, as I do now, volunteer my experience in defence of an ingredient, which I think truly valuable in the science of healing. It is not to be expected that *Digitalis* should operate as an universal panacea; nor have its advocates ever annexed to it the character of infallibility. They have told us, and they have told us truly, that *Digitalis will lesson the action of the heart;* and they have, therefore, very properly recommended it in diseases, in which the action of that organ was inordinately increased; and I am fully persuaded, that no man will ever abandon the use of *Digitalis,* who has given it a judicious and patient trial. I have known it administered *in the dose of one fourth of a grain every six hours, and to a robust man labouring under pulmonary inflammation.* In such contemptible doses it is impossible for it to effect any thing. The practitioner must be disappointed; the plant must lose its credit. But let the *Digitalis* be administered every two or three hours, in the dose of *twenty drops of the saturated tincture,* or in *one grain of the powder properly preserved,* in a short space of time the heart will come under its influence, and the blood will be sent through the lungs with diminished velocity. Again, I have heard of *Digitalis* being exhibited three or four times a day, and in the dose of three or four grains, without any sensible effect: this needs no comment. The plant must have lost is efficient qualities; and it is impossible for any man, to benefit society, or add to the stock of medical literature, who experiments in this slovenly manner.

I do not pretend to measure with exactness the precise quantity of *Digitalis* which may be necessary to influence the heart, and produce its full effect; but I know, that in ninety-nine cases of the hundred, the dose I have mentioned will be sufficient for this purpose. I also know, that when the urgency of the case demands it, I can, in twentytwenty-four hours, exhibit twelve grains of the powder, not only with the most perfect safety, but with the most striking advantage. When it is necessary speedily to change the system with *Digitalis,* and when the quantity above-mentioned is daily to be administered, it must be given in small, and frequently repeated doses. I need not observe, that under those circumstances, frequent visits to the bed-side of the patient are indispensible. The proper dose of any medicine is precisely that quantity which will produce the desired effect. We all know that *mercurial unction* will, sooner or later, influence the system; and yet no man can tell the number of drachms or ounces, which may be necessary to effect this change. *Digitalis* has been, and it will, I think, ever continue to be, a fashionable remedy in all the various stages of what is called *hectic fever*; and therefore it will be frequently administered under circumstances of disorganization, when no power could possibly preserve life, but the great Artist who first imparted it. If this paper should ever occupy the attention of *Harletonensis,* let me implore him, for the sake of humanity, to give *Digitalis* in *pleuritis, peripneumonia, phthisis* in its early stages, *mania,* in *general* and *local* dropsies; and, when, with due caution, he has given it a candid and a patient trial, let him tell me if he can abandon its use.

So much on the subject of *Digitalis.* I shall now very briefly notice the other articles which Harlestonensis has dismissed with marks of his disapprobation.

Has he ever employed the *uva ursi* in *diseases of the bladder and urinary passages?* and more especially, has he ever tried it in *gonorrhæa?* Has he ever administered the *lichen islandicus,* as a nutritive mucilage, in cases of *phthisis* combined with *digitalis,* and the well-known *myrrh mixture* of the late Dr. *Griffith?* Has he ever consulted Dr. *Marcet's* Report of the *oxyd of bismuth,* in the Memoirs of the London Medical Society? or has he ever perused the pages from the elegant pen of Dr. *Bardsley?* If he has not, I earnestly request that he may; that he may try the *oxyd* of the quality, in the form prescribed, and in the diseases pointed out, by that justly celebrated physician.

Has Harlestonensis ever paid any attention to the very excellent effects of distilled or soft water, in *affections of the stomach, accompanied by indigestion?*

By these queries, I am solicitous to fix Harlestonensis's attention to subjects, which he has not considered at all, or or very superficially; and before he banishes *mercury* from the class of remedies to be employed in *Typhus*, I would have him consult the writings, and pause upon the experience, of *Rush*, *Hamilton*, and *Jackson*.

I am, &c.

* GEORGE MOSSMAN.

*Bradford, Yorkshire, Sept.* 12, 1808.

---

*P. S.* In your last Number, there are two communications from Harlestonensis. The latter, addressed to "S. Strowger," and couched, as I think, in terms not the most becoming. The stateliness which he has assumed, and his affectation of treating his adversary with contempt, are never creditable qualities in a man of science, and very certainly they never bear with them any sort of conviction. The features of an unforgiving rivalship are too visibly marked in every line; and I really think that Mr. *Taylor* does not compliment either his own comprehension, or that of his Friends, by affecting not to understand the paper of Mr. *Strowger*; nor am I at all disposed to admit of Mr. *Taylor*'s claim of superiority, over his opponent. On the face of Mr. *Strowger's* paper, it is sufficiently plain to a very common understanding, that he means to recommend two fashionable, and, as he thinks, two very valuable articles of the *Materia Medica*. I know nothing of the parties, but I cannot help lamenting that any narrow jealousy should obtrude itself upon discussions so truly interesting.

* The Editors are much gratified by Dr. Mossman's resumption of his communications, as they have always been fully sensible of their value.

